# Engineering of Chitosan-Hydroxyapatite-Magnetite Hierarchical Scaffolds for Guided Bone Growth

**DOI:** 10.3390/ma12142321

**Published:** 2019-07-20

**Authors:** Alessandro Pistone, Daniela Iannazzo, Consuelo Celesti, Elpida Piperopoulos, Deepu Ashok, Arianna Cembran, Antonio Tricoli, David Nisbet

**Affiliations:** 1 Department of Engineering, University of Messina, Contrada Di Dio, I-98166 Messina, Italy; 2 Laboratory of Advanced Biomaterials, Research School of Electrical, Energy and Materials Engineering, Australian National University, Canberra ACT 2601, Australia; 3 Nanotechnology Research Laboratory, Research School of Electrical and Energy Engineering, Australian National University, Canberra ACT 2601, Australia

**Keywords:** chitosan/hydroxyapatite, osteoconduction, tissue engineering, magnetite

## Abstract

Bioabsorbable materials have received increasing attention as innovative systems for the development of osteoconductive biomaterials for bone tissue engineering. In this paper, chitosan-based composites were synthesized adding hydroxyapatite and/or magnetite in a chitosan matrix by in situ precipitation technique. Composites were characterized by optical and electron microscopy, thermogravimetric analyses (TGA), x-ray diffraction (XRD), and in vitro cell culture studies. Hydroxyapatite and magnetite were found to be homogeneously dispersed in the chitosan matrix and the composites showed superior biocompatibility and the ability to support cell attachment and proliferation; in particular, the chitosan/hydroxyapatite/magnetite composite (CS/HA/MGN) demonstrated superior bioactivity with respect to pure chitosan (CS) and to the chitosan/hydroxyapatite (CS/HA) scaffolds.

## 1. Introduction

The recent advances in nanomaterial fabrication methodology have dramatically improved the tissue-implant interface, offering alternative treatment options for patients affected by bone injuries or diseases [1,2,3]. To date, the most significant achievement has been in engineering sophisticated biomimicry within the implants that accelerate osteo-induction, -conduction and -integration. This has only been possible through the nanoscale engineering of the cell-biomaterial interaction, which is revolutionizing modern implant therapy [4,5,6]. However, many limitations remain, with the most significant being the dependence of implant success on the quality and quantity of bone in the recipient site [7]. In the field of dental implant, bone loss can also be related to several systemic and periodontal diseases, trauma and tumors, thus constituting a major challenge for achieving good long-term osteo-integrated implants [8]. Pathophysiologically, three-dimensional alveolar bone resorption occur as early as six months after tooth loss or extraction, thus posing a significant challenge for predictable implant placement. For these reasons, the reconstruction of the resorbed alveolar ridges has been a goal for clinicians in order to optimize outcomes of oral implant placement [9,10]. Different strategies, such as bone-grafting techniques, alveolar distraction osteogenesis and guided bone regeneration (GBR), have been used to reconstitute the lost bone and to facilitate implant integration and maintenance during the functional loading [11,12,13]. Among these techniques, GBR is most commonly employed for alveolar bone reconstruction and the treatment of peri-implant bone deficiencies. In principle, GBR uses barrier membranes that are able to exclude nonosteogenic cell populations from the surrounding soft tissues and to promote the survival and maintenance of slower-growing cells (osteoprogenitor cells) necessary for bone regeneration [14,15]. By the deployment of membranes to provide suitable areas for osteoprogenitor cells, their recruitment, proliferation, and differentiation towards osteoblastic specification are being promoted; this is essential for ossification [16]. The membranes used in GBR can be roughly divided into two types—bioabsorbable and non-resorbable membranes [17,18]. The non-resorbable membranes include expanded polytetrafluoroethylene, high-density polytetrafluoroethylene, and titanium-reinforced high-density polytetrafluoroethylene membranes [19]. However, even when these membranes have demonstrated excellent biocompatibility and good bone regeneration, their efficacy is challenged by soft tissue dehiscence, leading to chronic infection and ultimately revision surgery [20]. For this reason, bioabsorbable membranes have been investigated, with the current biological challenge being an ability to match resorption time with the rate of ossification [21]. The primary bioabsorbable membranes that are utilized can be classified into natural and synthetic polymers and composites [17,22]. The recognized requirements of such membranes are: Ease of manufacturability and scalability, possess hierarchical porous morphology to facility cells infiltration, remodeling and angiogenesis, adequate structural integrity that is partially critical for large defects, and they may induce osteoinduction and conduction [23]. Among the different biodegradable polymers deployed, chitosan as a natural polysaccharide, has shown significant promise, due to its inherent biocompatibility, biodegradability, and the ability for the thermoreversible hydrogel to fill irregular defects that are desirable for bone tissue engineering applications [24,25]. More interestingly, chitosan is often formed into a composite structure with calcium phosphate (CaP) to produce a useful adjuvant material (bone cement) with improving mechanical properties compared to chitosan hydrogel alone [26]. However, hydroxyapatite has been demonstrated to have superior osteoconductive properties to CaP, with a slower dissolution rate encouraging prolonger ossification [27,28,29]. When HA is incorporated with chitosan, it has been demonstrated to overcome some limitations of commonly employed bone cement that are inherently brittle and are difficult to fabricate in specific shapes [30,31].

Here, we have designed an HA/chitosan hierarchical scaffold, that is easily synthesizable in desirable geometries as a bioabsorbable membrane for implant therapy. The efficacy of the scaffold has been further increased by the inclusion of crystalline iron oxide nanoparticles. These were incorporated to increase the performance of the scaffolds, thanks to the super magnetic and ferromagnetic properties of iron oxide used in biomedical applications, such as magnetic resonance imaging, hyperthermia, and as drug delivery systems [32,33,34,35]. On this basis, we have investigated the ability of our chitosan/hydroxyapatite/magnetite (CS/HA/MGN) composites crosslinked with genipin as potential biomaterials that may induce osteoinduction and osteointegration. We observe that these materials are biocompatible with primary osteoblast cells. Remarkably, the inclusion of magnetite significantly increases the number of osteoblast cells that infiltrate the hierarchical scaffold and, more importantly for implant therapy, increases the number of viable osteoblast cells at the scaffold interface. Our findings show, for the first time, a facile in situ precipitation method to rapidly form superior guided bone regeneration scaffolds, demonstrating their in vitro cytocompatibility and their ability to promote osteoblast maintenance and differentiation.

## 2. Materials and Methods

### 2.1. Materials

Chitosan, acetic acid, hydroxyapatite, calcium nitrate tetrahydrate, ammonium phosphate dibasic, iron (II) chloride, iron (III) chloride, and ammonia solution were purchased from Sigma–Aldrich; genipin was purchased from Carbosynt. Detailed information about reagents and solvents are available in Appendix A. All reagents and solvents were used without further purification. 

All x-ray diffraction (XRD) experiments were performed at room temperature with a Bruker D8 Advance diffractometer (Bruker, Karlsruhe, Germany) using a Bragg–Brentano theta-2theta configuration, with Cu Ka radiation (40 V, 40 mA). XRD patterns were collected in the range 10–80° with a step of 0.2°/s. Diffraction peaks were compared with those in the Joint Committee on Powdered Diffraction Standards (JCPDS) database. Environmental Scanning Electron Microscopy (SEM) was conducted at room temperature on a FEI Quanta 450 FEG instrument (Thermo Fisher Scientific, Hillsboro, OR, USA) operating at 15 kV, using a Secondary Electron (SE) sensor. The Energy Dispersive X-ray (EDX) analysis was performed with an Octane Plus Silicon Drift Detector (Ametek, Berwyn, PA, USA), equipped with a 30 mm^2^ Super Ultra Thin Window (SUTW). FeK mapping analysis was also executed, using an image resolution of 256 × 200 pixels and a dwell time (time to collect EDX counts at each pixel in the collection region) of 200 μs. Mapping acquisition time was set at 60 min. A TAQ500 instrument (TA Instruments, New Castle, DE, USA) was used for thermogravimetric analyses (TGA) from 100 to 700 °C, with a rate of 20 °C per minute under air atmosphere. Optical images were recorded at room temperature by means of a Hirox digital microscope mod. KH8700 (Hirox, Tokyo, Japan) by mounting a MX(G)-5040Z lens. Fluorescence microscopy analyses were performed at room temperature by an Inverted Laboratory Microscope, Leica DM IL LED (Leica, Wetzlar, Germany).

### 2.2. Methods

#### 2.2.1. Synthesis of CS Samples

In this process, 240 mg of chitosan powder was dissolved in an aqueous solution of 2% acetic acid for 30 min at 45 °C. Afterwards, 24 mg (0.1 mmol) of genipin or 0.33 mg was slowly added into the mixture. The hydrogels were then rinsed with deionized water and dried in a baker at 37 °C for 24 h at the vacuum drying pressure of 65 mbar for subsequent characterizations.

#### 2.2.2. Synthesis of CS/HA Samples

In the process, 240 mg of chitosan was dissolved in an aqueous solution of 2% acetic acid for 30 min at 45 °C. Subsequently, 1.56 mmol of Ca(NO_3_)_2_·4H_2_O and 0.5 mmol of (NH_4_)_2_HPO_4_ was added under vigorous agitation. The solution was stirred for 30 min until the calcium and phosphate salts were entirely dissolved. After this, 24 mg (0.1 mmol) of genipin was slowly added into the mixture. The formed CS/HA composite was treated with a 2% NH_3_ solution for 1 h at room temperature and then was rinsed with deionized water until reaching pH 7. The formed hydrogel was dried in a baker at 37 °C for 24 h at the vacuum drying pressure of 65 mbar for subsequent characterizations. CS/HA sample starting from HA powder (240 mg) incorporated in chitosan during the cross-linking step was also synthesized.

#### 2.2.3. CS/HA/MGN Sample

The same process as for the CS/HA samples above was followed until the calcium and phosphate salts were completely dissolved. Then, 24 mg (0.1 mmol) of genipin was slowly added into the solution that now also contained FeCl_2_ (0.04 mmol) and FeCl_3_ (0.09 mmol) to produce the CS/HA/Fe_3_O_4_ composite scaffolds. These were subsequently treated with a 2% NH_3_ solution for 1 h at room temperature and then rinsed with deionized water until reaching pH 7. The composite was dried in a baker at 37 °C for 24 h at the vacuum drying pressure of 65 mbar for subsequent characterizations. Additionally, CS/HA/MGN sample was prepared from HA/Fe_3_O_4_ co-precipitated powders (240 mg) that were synthesized by a previously described procedure [34] and incorporated into chitosan during the cross-linking as above.

All the chitosan based samples were stored in a hermetic sealed pan at a temperature of 15 °C under relative humidities held constant by adding a beaker containing water inside the hermetic sealed pan. The formulations were periodically weighed to verify that no loss of water occurred. The composites were usually stored for a short period (1–3 days).

#### 2.2.4. In Vitro Cell Culture Studies

##### Primary Osteoblast Isolation and Culture Method

These studies conformed to the Australian National Health and Medical Research Council guidelines for use of animals in research and were approved by the Animal Ethics Committee. Nine-week old adult Swiss mice were culled, and their femur bone was dissected. The femur was minced and cleaned thoroughly in Phosphate Buffered Solution (PBS, Sigma–Aldrich, Milan, Italy) to remove the muscle tissue around it. The cleaned minces were then transferred to a 25 cm^2^ tissue culture flask (Thermo Fisher Scientific, Waltham, MA, USA) containing completed cell culture medium comprising of α-Minimum Essential Medium (α-MEM, Gibco, Scoresby, Australia), supplemented with 10% of fetal bovine serum (FBS, Gibco) and 1% of penicillin-streptomycin antibiotics (HyClone, Scoresby, Australia) and incubated and maintained in a humidified atmosphere (37 °C, 95% air, 5% CO_2_). Once reaching a confluent layer of cells, the cultures were mechanically detached from the flask bottom using a cell scraper and were seeded at a density of 1 × 10^4^ cells on both the PDL poly-_D_-Lysine (PDL, Merck, Sydney, Australia, 0.01 mg/mL in PBS) coated coverslips and the scaffolds.

##### Immunostaining of the Cells

After 2 days of culture, the cells were washed twice with PBS and fixed with 4% Paraformaldehyde (PFA, Sigma–Aldrich) for 10 min at room temperature followed by three PBS washes. Plates were then sealed with parafilm and stored in the fridge until immunofluorescence. Cells were permeabilized with PBS containing 0.1% Triton (Sigma–Aldrich; TPBS) for 10 min and washed in PBS three times before being incubated with a blocking solution containing 10% donkey serum (Merck) in TPBS for 30 min. The blocking solution was then aspirated and the samples were washed three times in TPBS washes. The cells were then incubated with osteocalcin (1:200, Abcam, Cambridge, UK) in 10% donkey serum and 0.1% TPBS overnight at 4 °C. After 24 h, the primary antibody solution was decanted and the cells were washed three times with PBS and incubated with the secondary antibody Alexa Fluor 488 (1:500, Abcam) in 5% donkey serum and 0.2% TPBS for 1.5 h at room temperature. Following this, cells were incubated for 5 min with Hoechst (1:5000, Life Technologies, Scoresby, Australia) and three PBS washes were performed prior to mounting the coverslips on a glass slide. Cells were then visualized under a fluorescence microscope (Inverted Laboratory Microscope, Leica DM IL LED, Wetzlar, Germany).

##### Sterilization and Osteoblast Culture on the Scaffolds

**Figure 1:**
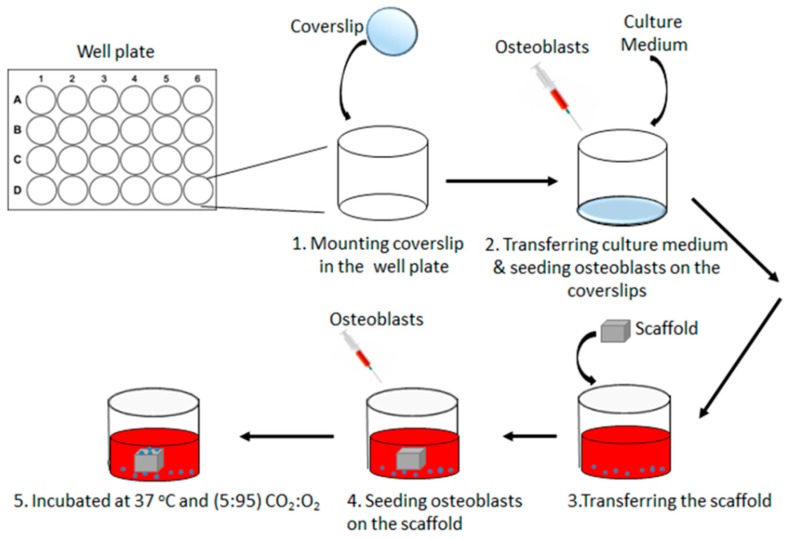
Schematic representation of the osteoblast seeding experiment.

Prior to the seeding, the scaffolds were sterilized by soaking in 1% penicillin-streptomycin antibiotics in PBS solution for 48 h in a humidified atmosphere (37 °C, 95% air, 5% CO_2_). After this, the scaffolds were soaked in the complete medium and incubated for a further 24 h. Coverslips were transferred to 24 well plates and washed with 70% ethanol followed by 20 min of exposure to ultraviolet radiation for sterilization. The 1 × 10^4^ primary osteoblasts were seeded onto the coverslips to analyze whether the scaffolds degrade into cytotoxic products over time and to explore the interaction of the biomaterials interface, a critical requirement to form GBR membranes. The sterilized scaffolds were then transferred to the well plates containing the coverslips. The 1 × 10^4^ primary osteoblasts were seeded on top of the scaffolds to probe the ability of the scaffolds to mechanically support cells and their migration. A well with a PDL coated glass coverslip and no scaffold in it was chosen as the control. A schematic of the cell-seeding experiment is shown in Figure 1.

##### Viability and Cytotoxicity Investigation of Osteoblasts in the Scaffold Environment

Cell viability assay was performed on the scaffolds and the coverslips after 48 h using the Live/Dead assay kit (Life Technologies). The optimal dye concentrations of Calcein AM, the live cell label and EthD-T, the dead cell label was optimized to 2 µM each. After 48 h the scaffolds were sliced and the sections and the coverslips were then used in image analysis under a fluorescence microscope (Inverted Laboratory Microscope, Leica DM IL LED) and the live cell and dead cell numbers were determined.

## 3. Results and Discussion

**Figure 2:**
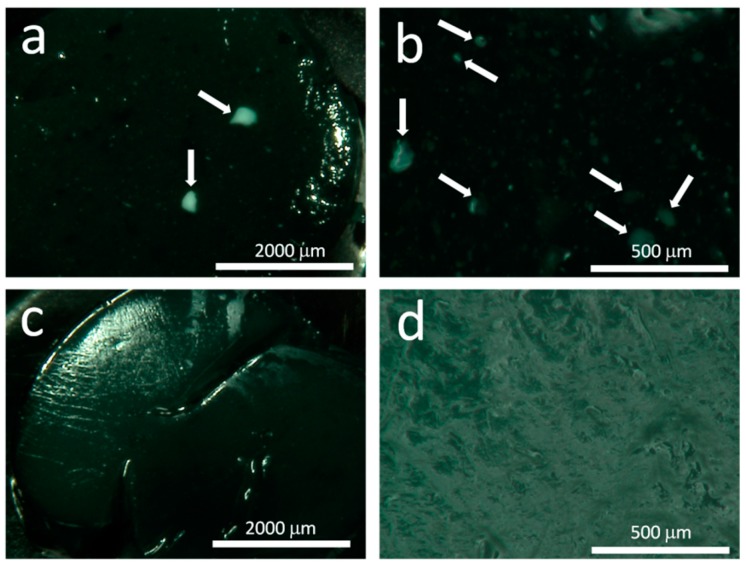
Optical images of chitosan/hydroxyapatite (CS/HA) composites prepared with HA powder (**a**, lower magnification, and **b**, higher magnification) and HA obtained by in situ precipitation (**c**, lower magnification, and **d**, higher magnification) (Images collected with Hirox digital microscope mod. KH8700 with a MX(G)-5040Z lens).

The chitosan/hydroxyapatite/magnetite (CS/HA/MGN) and chitosan/hydroxyapatite (CS/HA) composites were prepared by using an in situ precipitation method. This method was selected to ensure a superior homogeneous dispersion of the hydroxyapatite inside the chitosan matrix as highlighted in Figure 2. Large assemblies of HA (marked with arrows) were observed in the sample fabricated with HA powder, while a homogeneous distribution of HA appears in the sample prepared using our optimized in situ precipitation method.

**Figure 3:**
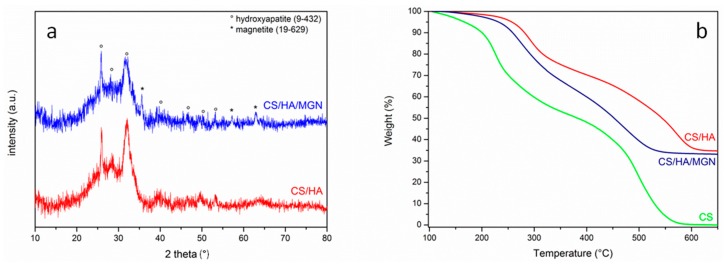
(**a**) x-ray diffraction (XRD) spectra of CS/HA and CS/HA/MGN (chitosan/hydroxyapatite/magnetite) composites; (**b**) thermogravimetric analyses (TGA) curves for CS, CS/HA and CS/HA/MGN samples. All experiments were performed under an air atmosphere.

The crystalline structures and thermal stability of the chitosan-based composed prepared by in situ precipitation method was investigated by XRD and TGA analyses (Figure 3). The hydroxyapatite [Ca_10_(PO_4_)_6_(OH)_2_] formation was verified by XRD analyses; as indicated in Figure 3a, the main characteristic lines of hydroxyapatite (JCPDS file no. 9-432) overlap well with the diffraction peaks of synthesized materials; in addition, the representative peaks of magnetite (JCPDS no. 19-629) at 2θ 35.5°, 57.2°, and 62.9° were also present.

In order to obtain information about the degree of crystallinity within the samples prepared via in-situ precipitation, the hydroxyapatite average crystallite size was calculated by Scherrer’s equation using the (002) reflection peak at 2θ 26°, which is well resolved and shows no interference. (Equation (1)):

*L* = (0.9⋅*λ*)/(*β*_002_·cos*θ*) (1)

where the *L* value represents the average crystallite size of the hydroxyapatite, *β*_002_ is the peak width at the half maximum of the (002) peak expressed in radians, λ is the wavelength of the X-ray radiation (Cu Kα radiation, *λ* = 0.15418 nm) and *θ* (radians) is the angular position of the (002) peak. The conversion of *β*_002_ value from degrees to radians is obtained using Equation (2):

*β*_002_(rad) = *β*_002_(2theta degree)⋅*π*/180 (2)

The crystallinity, noted by *Xc*, that corresponds to the fraction of the crystalline hydroxyapatite phase in the investigated volume of the powdered sample was calculated using an empirical relation between *Xc* and the *β*_002_, according to Equation (3):

*Xc* = (*K*/*β*_002_)^3^ (3)

where *Xc* is the crystallinity degree, *β*_002_ is the full width of the peak at the half intensity of the (002) reflection in 2theta degree and *K* is a constant set at 0.24 [36,37].

Data reported in Table 1 show that the chitosan-based systems entrapped the hydroxyapatite having low or medium values of crystallinity and/or nanosized crystallites, ensuring a high metabolic activity. This is important, as the bioactivity of hydroxyapatite, in terms of bioresorption via chemical bonding with surrounding hard tissues, depends strongly on its crystallinity and particle size distribution [38,39].

Thermogravimetric analysis was performed on both CS, CS/HA, and CS/HA/MGN systems. All samples were pre-treated at 100 °C until a constant weight was achieved before they were subsequently heated up to 700 °C with a rate of 20 °C/min under air flow (Figure 3b). Hydroxyapatite had no weight loss in the temperature range investigated. As expected, the CS component of the scaffold was completely oxidized, and that was evident by the two degradation profile; the first between 200 and 300 °C and second between 450 and 580 °C. The presence of hydroxyapatite was shown to shift the first degradation profile to higher temperatures (between 250 and 350 °C) indicating a better thermal stability of the chitosan matrix in the presence of inorganic compounds which probably hinder the thermo-oxidation of organic matrix [40]. The residual weight at a temperature above 600 °C of 33–34 wt% indicates the hydroxyapatite or hydroxyapatite/iron oxides loading in the chitosan-based composites. Furthermore, in the CS/HA/MGN samples, iron oxides display their known catalytic properties in combustion reactions, catalyzing the degradation of chitosan, with the weight loss in the higher temperature range being completed at 530 °C, while in the CS and CS/HA systems this was extended to 580 and 610 °C, respectively. Importantly, the TGA results confirm that the chitosan overall structure was preserved during the in situ precipitation of hydroxyapatite or hydroxyapatite/iron oxides, as observed by the similar weight loss profiles of the composites with respect to pure chitosan.

**Figure 4:**
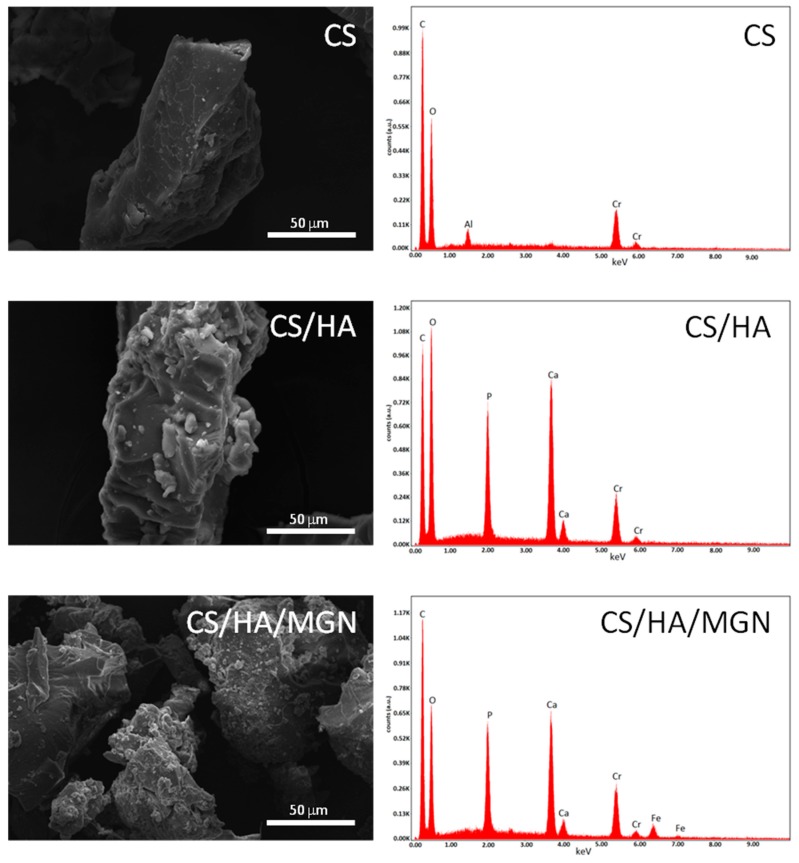
Representative Scanning Electron Microscopy/Energy Dispersive X-ray (SEM/EDX) analysis of CS, CS/HA, and CS/HA/MGN composites.

**Figure 5:**
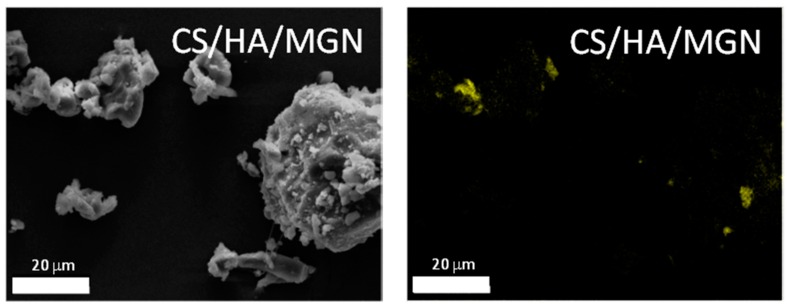
Mapping analysis performed on CS/HA/MGN composite.

Next, we investigated the surface morphology and homogeneity in the structure of the synthesized composites via field emission scanning electron microscopy (FEG-SEM). Prior to SEM analysis, the samples were sputter coated with a chromium layer. SEM revealed that the cross-linked chitosan had a smooth surface with a layered structure (Figure 4). The addition of hydroxyapatite and/or hydroxyapatite/iron oxide in the formulation, lead to the appearance of agglomerates on the surface of chitosan without any change on the overall and layered structure of the polymer. EDX analyses, shown in Figure 4, confirm the presence of hydroxyapatite or iron oxides in the composites. Furthermore, mapping analysis performed on CS/HA/MGN composites showed that the iron ions (yellow spots) were homogeneously distributed in the composites with an atomic ratio Fe/Ca close to 0.19 (Figure 5). This value, coupled with the TGA result, showing a residual inorganic mass in the CS/HA/MGN sample of 33 wt%, allow us to determine the composition of CS/HA/MGN as indicated in Table 2.

**Figure 6:**
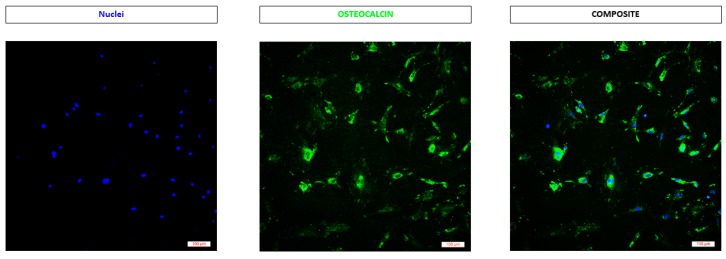
Immunostaining of the osteoblasts. Fluorescence images confirms the positive staining of osteocalcin.

Osteoblasts are the group of cells that are responsible for bone formation and are responsible for the secretion of the osteocalcin protein. Therefore, to confirm the purity of our primary osteoblast culture, the cells were incubated with the osteocalcin (OC) primary antibody followed by staining the nuclei with Hoechst solution. Fluorescence microscope images confirmed the purity of our culture with 88% of the cells being positive for osteocalcin (OC) (Figure 6).

**Figure 7:**
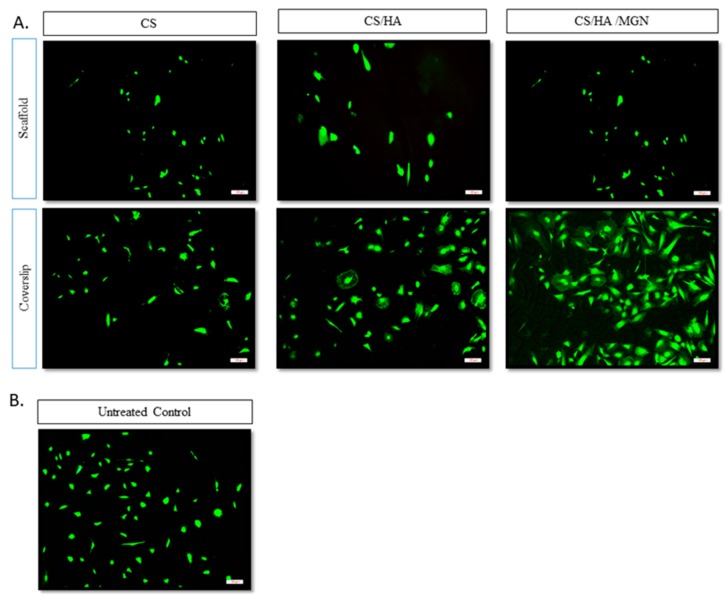
Cell viability in the scaffold environment. (**A**) Live/dead assay of primary osteoblasts cultured for 48 h on the scaffold and the scaffold-mounted coverslips. (**B**) Live/dead assay of the untreated control after 48 h of culturing the cells. The images show live (green) cells. Images were taken with 10× objective. Scale bar = 100 µm.

Next, to test the suitability of these materials for guided bone growth, cell viability was explored to evaluate the effect of the scaffold environment on cell behavior. This becomes particularly challenging in bone tissue engineering, as it has proven difficult to engineer suitable hierarchical materials that can support the phenotypic expression of osteoblasts and chondrocytes. Here, we have overcome this to show the presence of a high density of live osteoblasts on all scaffolds after 48 h in culture, verifying that all the surfaces support the cell attachment and proliferation (Figure 7A). The enumeration of the cell cultures in vitro indicates that the CS/HA/MGN scaffold promotes better cell infiltration in comparison to other groups (Figure 7A).

**Figure 8:**
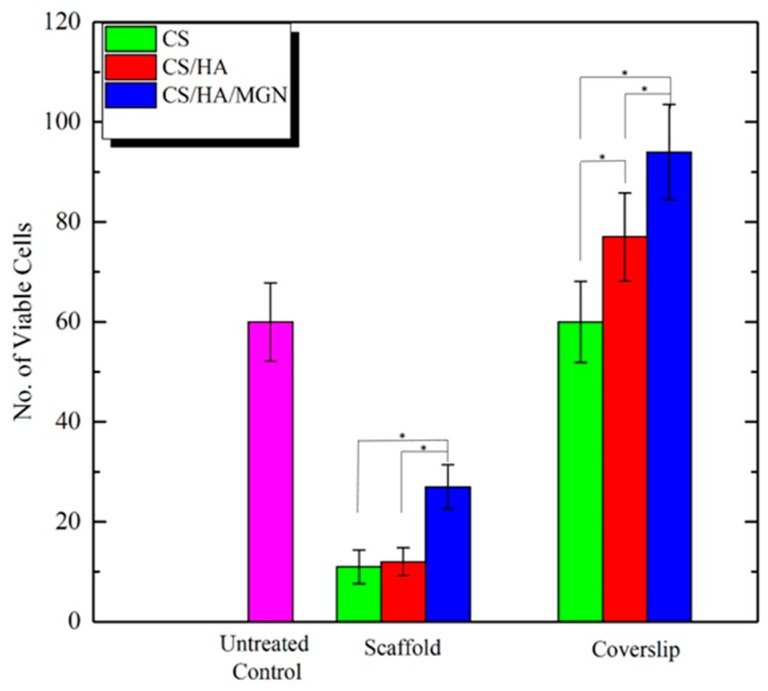
Cell viability in the scaffold environment. Live cells quantification. Plot showing the average quantified cells on the different scaffolds and test samples (error bars show an average of three measurements and * indicates *p* < 0.05 in an unpaired student *t*-test.).

We evaluated the biocompatibility of the cells in the immediate microenvironment of the scaffolds to conclude how osteoblasts interact with the tissue-implant interface. Cytotoxicity analysis was performed by Live/Dead staining on the coverslips, with cells that were on the surface of the coverslip directly underneath the scaffold being characterized. Fluorescence images show the presence of active osteoblasts on all the coverslips (Figure 7) confirming the non-toxic nature of the scaffolds over time, with the coverslip corresponding to CS/HA/MGN with the highest number of cells due to our novel inclusions of magnetite and the positive influence of its super magnetic and ferromagnetic properties on osteoblast maintenance and differentiation [34]. However, it is also possible that the inclusion of magnetite increases the overall wettability of the scaffolds and subsequently protein adsorption and conformation and also it could affect the gene signaling pathways, or that the tiny magnetic moments created by them could influence the microenvironment around the materials, which resulted in higher cell growth on the scaffolds and the coverslips [41,42,43]. The untreated control and the coverslips with CS had a comparable number of cells on it (Figure 8). Importantly, we have demonstrated the bioactivity of the CS/HA/MGN scaffold to be superior to that of CS or CS/HA scaffolds opening further improvement in cell proliferation by application of the external magnetic field. 

## 4. Conclusions

Here, composites based on hydroxyapatite and/or magnetite in chitosan matrix were synthesized by in situ precipitation technique and using genipin as the crosslinking chitosan agent. Morphological and chemical characterizations confirmed the homogenous distribution of crystalline hydroxyapatite and magnetite in the biopolymeric matrix. In vitro cell culture studies revealed that all the scaffold surfaces support cell attachment and proliferation, confirming the non-toxic nature of the scaffolds over time. In particular, the composite based on chitosan/hydroxyapatite/magnetite demonstrated superior bioactivity, making it a suitable and promising material for guided bone growth.

## Figures and Tables

**Table 1:** Average crystallite size and crystallinity degree of hydroxyapatite in the chitosan-based systems.

Sample	Full-Width at Half Maximum of (002) Peak (FWHM)	Crystallinity	Average Crystallite Size (L)
	(2θ)	(Xc)	(nm)
CS/HA	0.32	0.421875	25.26
CS/HA/MGN	0.44	0.162284	18.46

**Table 2:** Composition of chitosan-based samples as calculated by thermogravimetric analyses (TGA) and Energy Dispersive X-ray (EDX) analyses.

Sample Code	Composition (wt %)		
	Chitosan	Hydroxyapatite	Magnetite
CS	100	//	//
CS/HA	66	34	//
CS/HA/MGN	67	28.8	4.2

## Appendix A

Reagents and solvents details: Chitosan (CAS number 9012-76-4, Supplier code 448877 Sigma–Aldrich), genipin (CAS number 6902-77-8, Supplier code FG30976 Carbosynt), acetic acid (CAS number 64-19-7, Supplier code 33209-1L-M Sigma–Aldrich), hydroxyapatite (CAS number 12167-74-7, Supplier code 677418 Sigma–Aldrich), calcium nitrate tetrahydrate (CAS number 13477-34-4, Supplier code C1396 Sigma–Aldrich), ammonium phosphate dibasic (CAS number 7783-28-0, Supplier code 215996 Sigma–Aldrich), iron (II) chloride (CAS number 7705-08-0, Supplier code 372870 Sigma–Aldrich), iron (III) chloride (CAS number 7758-94-3, Supplier code 157740 Sigma–Aldrich), ammonium hydroxide solution (CAS number 1336-21-6, Supplier code 221228-1L-A Sigma–Aldrich).
